# Bilateral Leydig Cell Hyperplasia: A Rare Cause of Postmenopausal Hirsutism

**DOI:** 10.1155/2022/8804856

**Published:** 2022-02-12

**Authors:** S. Pathmanathan, S. D. N. De Silva, M. Sumanatilleke, D. Lokuhetty, U. V. V. Ranathunga

**Affiliations:** ^1^Diabetes and Endocrinology Unit, National Hospital of Sri Lanka, Colombo, Sri Lanka; ^2^Department of Pathology, Faculty of Medicine, University of Colombo, Colombo, Sri Lanka

## Abstract

**Background:**

Postmenopausal hirsutism could be due to a myriad of causes, including ovarian and adrenal tumours, ovarian hyperthecosis, exogenous androgens, and Cushing's syndrome. We report a patient who was found to have a rare cause of postmenopausal hirsutism. *Case Presentation*. A 64-year-old postmenopausal woman with a history of hypertension, thyrotoxicosis, and poorly controlled diabetes on multiple oral hypoglycaemic agents presented with gradual onset progressive excessive hair growth without any virilizing features. On examination, she did not have Cushingnoid features or clitoromegaly. Her hirsutism was quantified with Ferriman–Gallwey score which was 9. Her biochemical evaluation showed elevated testosterone levels with normal DHEAS, ODST, 17-OHP, and prolactin. Low-dose dexamethasone suppression test did not suppress testosterone more than 40%. Contrast-enhanced CT of the adrenal and pelvis did not show any adrenal or ovarian mass lesions. Transvaginal ultrasound scan showed bilateral prominent ovaries only. Combined adrenal and ovarian venous sampling was carried out to localize the source of excess androgen, but only the left adrenal vein was successfully cannulated which showed suppressed testosterone level compared to periphery. The patient underwent total abdominal hysterectomy and bilateral salphingo oophorectomy, and her testosterone level normalized postoperatively. Her glycaemic control improved. Histology showed evidence of bilateral diffuse ovarian Leydig cell hyperplasia.

**Conclusion:**

Evaluation of postmenopausal hirsutism needs careful history and examination followed by biochemical evaluation and imaging. While adrenal and ovarian venous sampling can help to arrive at a diagnosis, it is a technically demanding procedure with low success rates even at centers of excellence. Therefore, in such situations, bilateral oophorectomy may be the best course of action which will give the histological confirmation of the diagnosis. Successful treatment of hyperandrogenism can result in improvement of glycaemic control. Bilateral diffuse Leydig cell hyperplasia is a rare but important cause of postmenopausal hirsutism.

## 1. Background

Postmenopausal androgen excess can present as hirsutism or frank virilization. Proper evaluation and management of this clinical entity is of paramount importance to improve the quality of life and psycho-social well-being of these patients. [1].

We report a case of postmenopausal hirsutism due to bilateral ovarian diffuse Leydig cell hyperplasia which is quite a rare entity.

## 2. Case presentation

A 64-year-old lady presented with a 5-year history of hirsutism. She entered menopause at the age of 45 years. She initially noted excessive hair growth involving the upper lips, chin, arms, and legs at the age of 59 years. There was progression of symptoms since the age of 61 years. However, there was no associated frontal balding, enlarged muscle mass or deepening of voice. She attained menarche at the age of 12 years and had regular menstrual cycles until menopause. She did not have any subfertility or excessive hair growth during her reproductive period suggestive of polycystic ovarian syndrome. She is a diagnosed patient with type II diabetes mellitus for 12 years and was on maximum doses of three oral hypoglycaemic agents without any evidence of micro- or macrovascular complications. Deterioration of glycaemic control has been noted for the past 1 year. She is also a diagnosed patient with hypertension for 12 years and thyrotoxicosis due to Graves' disease for 2 years, on medical therapy with satisfactory control. She had delivered 4 children through normal vaginal delivery without any significant obstetric or neonatal complications. She was not on any androgenic drugs. She is a nonsmoker, and there was no significant family history of any malignancy.

On examination, her weight was 76 kg and height was 160 cm, and body mass index was 29.68 kg/m^2^. She had dark, coarse, terminal hair in androgen dependent areas with a Ferriman–Gallwey score of 9 (upper lip: 3; chin: 3; abdomen: 1; arm: 1; thighs: 1). There was no frontal balding or clitoromegaly. She did not have acanthosis nigricans, acne, or Cushingnoid features. She was clinically euthyroid with a pulse rate of 68 beats per minute and blood pressure of 130/80 mmHg.

Biochemical evaluation showed increased serum fasting testosterone levels (measured by the fully automated immunoassay analyzer ADVIA Centaur XP). Follicle-stimulating hormone (FSH) and luteinizing hormone (LH) levels were in the expected range for postmenopausal women. Basal 17-hydroxyprogesterone (17-OHP) level and dehydroepiandrosterone sulphate (DHEAS) level were normal. Hyperandrogenism was confirmed by repeating serum testosterone which was 176 ng/dL. Fasting blood sugar was 212 mg/dL, and HbA1c was 12.1 mmol/L (Table 1).

To evaluate the source of excess testosterone, we measured dexamethasone suppressed testosterone level and carried out ovarian and adrenal imaging studies. Serum testosterone level was checked after oral dexamethasone 0.5 mg 6 hourly for 48 hours. Serum testosterone level got suppressed by 36.9% (basal serum testosterone 176 ng/dL and after dexamethasone 111 ng/dL). Studies have shown that the majority of patients with nontumerous hyperandrogenism normalizes testosterone or suppress by more than 40% after low dose dexamethasone suppression [2]. Therefore, the possibility of an androgen secreting tumour was considered in our patient. Although nonsuppression of testosterone is thought to indicate an ovarian source, no studies of hyperandrogenism in postmenopausal women have been reported using this approach.

Transvaginal ultrasound scan showed prominent ovaries (right ovary: 3.5 cm*∗*2.1 cm; left ovary: 3.4 cm*∗*2.2 cm), and her endometrial thickness was 6 mm which is slightly above normal for postmenopausal women. Contrast-enhanced CT scan of the abdomen and pelvis showed normal bilateral adrenal glands and a left adenexial cystic lesion suggestive of a simple ovarian cyst. Her CA-125 level was 4.5 U/mL (normal < 30.2). At this point, we were not able to exclude an adrenal source of androgen excess reliably. Thus, bilateral oophorectomy could be a futile surgery if hyperandrogenism did not resolve after surgery due to a missed small adrenal tumour. Therefore, we decided it would be worthwhile going ahead with combined adrenal and ovarian venous sampling to confirm the source of androgen excess prior to surgery. Biochemical confirmation of successful catheterization of the ovarian veins was based on an estradiol ratio >2 and that of adrenal veins when the cortisol ratio was >2. A testosterone ratio of >2 was considered to imply a source of androgen production [3]. Unfortunately, only the left adrenal vein fulfilled the biochemical criteria for successful catheterization and the testosterone ratio of the left adrenal vein to femoral vein was 0.6, thus excluding the left adrenal gland as the possible source of testosterone excess.

**Figure 1:**
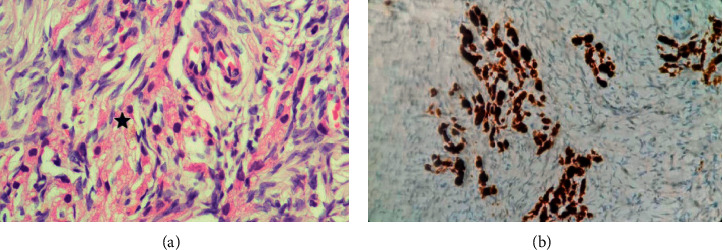
Histology of right ovary showing small clusters of Leydig cells, which has oval round nuclei with inconspicuous nucleoli and moderate eosinophilic cytoplasm (a, asterix, H&E, 400x). The Leydig cells display strong cytoplasmic staining with Calretinin (b, 400x). Histological appearance of the left ovary was similar.

**Figure 2:**
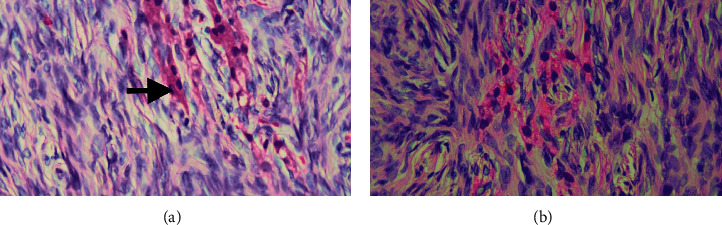
Rod-like structure resembling a Reinke crystal (a, arrow, H&E, 400x). Leydig cells with cytoplasmic vacuolation and brown lipofuschin pigment (b, H&E, 400x).

Although we could not definitively exclude the right adrenal gland as the possible source of excess androgen considering the negative imaging, we decided to refer her to surgery and she underwent total abdominal hysterectomy and bilateral salphingo oophorectomy. Total abdominal hysterectomy was done because her endometrial thickness was marginally high in the initial evaluation, and at her age, benefit of the procedure was considered to be more than any risk or adverse event. There were no surgical complications. On pathological examination, the right and left ovaries measured 40 × 30 × 17 mm and 50 × 30 × 23 mm, respectively. Both ovaries revealed a smooth outer surface and a homogenously tan white cut surface. There was no evidence of haemorrhage, cyst formation, or necrosis. Histological examination of bilateral ovaries revealed Leydig cells diffusely scattered within the normal appearing ovarian stroma (Figures 1 and 2). Cell clusters did not form large aggregated nodules leading to either significant ovarian enlargement or shape distortion. There was no evidence of nuclear atypia or increased mitotic activity. Similar cell clusters were also seen in the hilus. Androgen receptor immunostaining did not reveal any theca cells. Sertoli cells were not seen. On postsurgery day 1, total testosterone level came down to 15.27 ng/dL (normal range: 14–76). Her fasting blood sugar level was 85 mg/dL, and we were able to reduce the doses of oral hypoglycaemic agents. In 3 months of follow-up, her hirsutism showed a marked improvement.

## 3. Discussion

In this postmenopausal lady with clinical and biochemical hyperandrogenism with clear postmenopausal onset and normal menstruation and fertility prior to menopause, normal ODST, and no history of exogenous androgen exposure, only 3 differential diagnoses remain [4]: androgen-secreting adrenal tumours, androgen-secreting ovarian tumours (Sertoli–Leydig cells, granulosa-theca cells, hilus cells), and ovarian hyperthecosis/leydig cell hyperplasia.

Imaging studies like transvaginal ultrasound scan and contrast-enhanced CT can help in differentiation. If the imaging studies are negative, the following investigations can help in differentiation.

1. High DHEAS levels will support a diagnosis of adrenal tumour. However, in a study of 478 women with hyperandrogenism, 10 patients had DHEAS level more than 16.3 *μ*mol/L, and none of them were found to have an adrenal tumour [5].
2. Low dose dexamethasone suppression of serum testosterone can differentiate ovarian and adrenal neoplasms from nonneoplastic hyperandrogenism as testosterone level is suppressed by more than 40% in the latter but not in the former [2].
3. Combined ovarian and adrenal venous sampling is a technically challenging procedure and therefore is not routinely recommended. Even in centers of excellence where the procedure is done by an experienced radiologist, the success rate of venous catheterization has been quite low: all four veins in 27%, three veins in 65%, and two veins in 87% [3].
4. Ovarian androgen synthesis depends on LH stimulation while adrenal does not. Therefore, suppression of LH with GnRH analogues (GnRH-analog suppression test) leads to a reduction of testosterone level by more than 50%, if the ovary is the source of androgen excess. However, this test does not differentiate between ovarian hyperthecosis and virilizing ovarian tumours [6].

There are 3 main options for the management of nontumerous hyperandrogenism such as ovarian hyperthecosis or Leydig cell hyperplasia.

1. Anti-androgen-cyproterone acetate is a competitive inhibitor of testosterone at the level of androgen receptors.
2. As described above, GnRH analogues suppress LH, thus leading to suppression of ovarian androgen production.
3. Bilateral oophorectomy is the definitive treatment. Adrenal tumours are almost always visible on contrast-enhanced adrenal protocol CT. Therefore, if the adrenal CT is negative, bilateral oophorectomy may be the best therapeutic option.

Leydig cell hyperplasia is a rare cause of postmenopausal hirsutism. Review of the literature yielded only about 10 reported patients with this condition [7–14].

In postmenopausal women, the ovarian stroma is a source of testosterone [15]. However, ovarian hilar cells, which are morphologically indistinguishable from testicular Leydig cells, also secrete testosterone in the postmenopausal ovary. Hilar cells are predominantly seen in the ovarian hilum. Sometimes these cells can extend to the ovarian medullary stroma and such cells are called “Leydig cells of non-hilar type” [11]. Leydig cells are present in more than 80% of all female ovaries. These Leydig cells can undergo hyperplasia or can transform into a tumour. Distinction between the two entities is based on the histopathology depending on the pattern and size of the cell clusters. Hyperplasia is usually nodular but widely separated and diffuse. A nodule more than 1 cm is considered a tumour.

Etiopathogenesis of Leydig cell hyperplasia is not well defined. However, it could be due to autonomous origin or central stimulation due to high LH levels seen in postmenopausal women [12].

Ovarian stroma has two types of cells: female type granulosa and theca cells and male type Sertoli and Leydig cells. Ovarian hyperthecosis is a morphologic alteration of ovarian stromal cells so that they resemble luteneized theca cells. There are ample case reports of this entity [16]. In our patient, even though ovarian hyperthecosis comes in to the differential diagnosis, diffusely proliferating Leydig cells among normal stromal cells were favoured on histomorphology. Even though few Leydig cells were seen in the ovarian hilar region, there was no associated hyperplasia. This pattern of diffuse Leydig cell hyperplasia within the normal stroma has been described only in a very few patients previously.

High testosterone levels are associated with a lower risk of type 2 diabetes mellitus in men and a higher risk in women [17]. Possible physiological mechanism of testosterone-induced increased insulin resistance in women is testosterone-induced serine phosphorylation of insulin receptor substrate-1 (IRS-1). IRS-1 is a signaling protein which couples insulin receptor to the phosphoinositide-3-kinase (PI3K) signaling cascade, which is important in bringing about the biologic actions of insulin. Serine phosphorylation of IRS-1 leads to uncoupling of insulin receptor and PI3K, thus leading to insulin resistance [18]. This may be the explanation for the deterioration of glycaemic control of our patient with hyperandrogenism and improvement of glycaemic control after surgery.

## 4. Conclusions

Bilateral ovarian diffuse Leydig cell hyperplasia is a rare but important cause of postmenopausal hirsutism. Successful treatment of hyperandrogenism in postmenopausal women can lead to reduced insulin resistance and improved glycaemic control.

## Figures and Tables

**Table 1:** Relevant hormonal evaluation of the patient.

Investigation	Value	Reference range
Total testosterone	123 ng/dL	14–76 (adult female)
FSH	31.92 IU/L	23–116.3 (postmenopausal)
LH	26.33 IU/L	15.9–54 (postmenopausal)
Prolactin	5.8 mU/L	48–430 (postmenopausal)
9.00 am cortisol (ODST)	24 nmol/L	<50
DHEAS	1 *μ*mol/L	0.8–4.9
17-OHP	5.8 nmol/L	<6 excludes 21OHD
SHBG	53.3 mmol/L	13.5–71.4

FSH: follicle-stimulating hormone; LH: luteinizing hormone; ODST: overnight dexamethasone suppression test; DHEAS: dehydroepiandrosterone sulphate; 17-OHP: 17-hydroxyprogesterone; SHBG: sex hormone binding globulin, 21OHD: 21–hydroxylase deficiency.

## Data Availability

The data used to support the findings of this study are available on request.

## Abbreviations

17–OHP: 17-Hydroxyprogesterone
CA–125: Cancer antigen 125
CT: Computed tomography
DHEAS: Dehydroepiandrosterone sulphate
DL: Dyslipidaemia
DM: Diabetes mellitus
FSH: Follicle-stimulating hormone
HbA1c: Haemoglobin A1C
LH: Luteinizing hormone
ODST: Overnight dexamethasone suppression test
SHBG: Sex hormone binding globulin.
